# Comparison of Oral Microbial Profiles between Children with Severe Early Childhood Caries and Caries-Free Children Using the Human Oral Microbe Identification Microarray

**DOI:** 10.1371/journal.pone.0122075

**Published:** 2015-03-30

**Authors:** Chen Ma, Feng Chen, Yifei Zhang, Xiangyu Sun, Peiyuan Tong, Yan Si, Shuguo Zheng

**Affiliations:** 1 Department of Preventive Dentistry, Peking University School and Hospital of Stomatology, Beijing, China; 2 Central Laboratory, Peking University School and Hospital of Stomatology, Beijing, China; LSU Health Sciences Center School of Dentistry, UNITED STATES

## Abstract

**Objective:**

Early childhood caries (ECC) has become a prevalent public health problem among Chinese preschool children. The bacterial microflora is considered to be an important factor in the formation and progress of dental caries. However, high-throughput and large-scale studies of the primary dentition are lacking. The present study aimed to compare oral microbial profiles between children with severe ECC (SECC) and caries-free children.

**Methods:**

Both saliva and supragingival plaque samples were obtained from children with SECC (n = 20) and caries-free children (n = 20) aged 3 to 4 years. The samples were assayed using the Human Oral Microbe Identification Microarray (HOMIM).

**Results:**

A total of 379 bacterial species were detected in both the saliva and supragingival plaque samples from all children. Thirteen (including *Streptococcus*) and two (*Streptococcus* and *Actinomyces*) bacterial species in supragingival plaque and saliva, respectively, showed significant differences in prevalence between the two groups. Of these, the frequency of *Streptococcus mutans* detection was significantly higher in both saliva (p = 0.026) and plaque (p = 0.006) samples from the SECC group than in those from the caries-free group.

**Conclusions:**

The findings of our study revealed differences in the oral microbiota between the SECC and caries-free groups Several genera, including *Streptococcus*, *Porphyromonas*, and *Actinomyces*, are strongly associated with SECC and can be potential biomarkers of dental caries in the primary dentition.

## Introduction

Previous studies on early childhood caries (ECC) have focused on *Streptococcus mutans*, *Actinomyces*, and *Lactobacillus* [1,2,3]. ECC has become a prevalent public health problem among preschool children globally, particularly in China. According to the third national oral health epidemiological survey conducted in China in 2005, the prevalence rate of dental caries among 5-year-olds was 66%, which was significantly higher than the average in other countries [6]. The bacterial microflora is considered to be an important factor in the formation and progress of dental caries. Researchers have explored the bacterial microbiota in dental plaque samples to investigate the etiology of severe ECC (SECC) using methods such as denaturing gradient gel electrophoresis, pyrosequencing analysis, and cultivation [1,2,3].

More than 700 bacterial species or phylotypes exist in the oral cavity, approximately 35% of which have not been cultivated [4]. Conventional microbiological approaches that rely on cultivation for the detection of microorganisms in the oral cavity are not sufficient for such comprehensive and intensive monitoring. These time-consuming techniques require many specialized and complex growth media and yet capture only a small fraction of the oral microbiota [1,5,6]. Therefore, several oral bacterial species remain undetected.

The Human Oral Microbe Identification Microarray (HOMIM), which targets approximately 300 predominant oral bacterial species that include cultivable and not-yet-cultivated phylotypes (HOMIM home page: http://mim.forsyth.org/), has recently been used to determine bacterial profiles and microbial diversity in the oral cavity and compare these between healthy individuals and those with oral diseases such as periodontitis [6,7]. In the present study, we used HOMIM with the aim of comparing the bacterial profiles in saliva and supragingival plaque samples between children with SECC and caries-free children to investigate the etiology of caries in the primary teeth. The data obtained may help in defining differences in the oral microbiota between children with SECC and caries-free children, identify potential biomarkers of ECC in the primary dentition, and further improve our understanding of this complex infectious disease.

## Materials and Methods

### Ethics Statement

Written informed consent was obtained from the parents of all children included in this study. The study design, protocol, and informed consent forms were approved by the Ethics Committee of Peking University Health Science Center (PKUSSIRB-2013060).

### Clinical Methods

A total of 40 Chinese children aged 3 to 4 years (39 to 50 months), including 20 caries-free [decayed, missing, filled surfaces (DMFS) index = 0] children and 20 children with SECC (DMFS ≥ 4 for 3-year-olds, DMFS ≥ 5 for 4-year-olds; Table 1), were included in this study. The definitions and diagnoses of dental caries and SECC were according to the criteria of the World Health Organization [8]. Children with no clinical signs of early caries or white spots were considered to be free of caries. The first molar had not erupted in any of the 40 children, and none of the patients exhibited salivary gland diseases or systemic diseases. No patients had consumed antibiotics within 4 weeks before the study. Samples were obtained only after obtaining informed consent from the children and their parents.

**Table 1:** Demographic and clinical characteristics of the study population.

Characteristic	Severe ECC n = 20	Caries-free n = 20	P value
Mean age (months) (mean ± SD)	43.9 ± 3.2	44.4 ± 3.7	0.62 ^a^
No. (%) male	9 (45)	10 (50)	0.759 ^a^
Caries status			
DMFS score (mean ± SD)	11.1 ± 5.6	0	<0.0001 ^a^
Low	4	0	
Medium	10.4	0	
High	24	0	

^a^ Nonparametric Mann–Whitney U test

DMFS: decayed, missing, and filled tooth surfaces

ECC: early childhood caries

All subjects were instructed to refrain from eating or drinking from 2 h before sampling. Stimulated whole saliva samples were collected in 5-mL sterile Eppendorf microcentrifuge tubes. Supragingival pooled plaque samples were obtained from the noncarious enamel surface of each tooth, including the anterior and posterior teeth, and each sample obtained from the same individual was placed in a 1.5-mL microcentrifuge tube containing 1 mL of TE (50 mM Tris-HCl, 1 mM EDTA; pH 7.6). These samples were immediately frozen at −20°C and stored at −80°C until further use.

### DNA Isolation and Amplification

Bacterial DNA was extracted from saliva and supragingival plaque using the TIANamp Bacteria DNA Kit (Tiangen Qiagen, Hilden, Germany). The lysis procedure was a modification of the conventional procedure [2,3]. All our samples were treated with a cocktail lysis buffer containing mutanolysin, proteinase K, and a lysozyme. The extracts were stored at −20°C until further use. 16S rRNA genes were amplified by polymerase chain reaction (PCR) from the bacterial DNA isolated from the saliva and dental plaque samples using universal 16S rRNA primers. Briefly, two separate PCR assays were performed using either the forward primer 5′-CCA GAGTTT GAT YMT GGC-3′ and reverse primer 5′-GAA GGA GGT GWT CCA RCC GCA-3′ or the forward primer 5′-GAC TAG AGT TTG ATY MTG GC-3′ and reverse primer 5′-GYT ACC TTG TTA CGA CTT-3′ [6]. The results of PCR amplification were examined by electrophoresis in a 1% agarose gel. The two parallel PCRs were combined and the products were purified using the TIAN quick Midi Purification Kit (Tiangen).

### Microarray Analysis

For HOMIM 16S rRNA gene microarray analysis, purified DNA samples (two plaque samples from the caries-free group were excluded because of poor quality of DNA) were sent to the MIM Core Facility at the Forsyth Institute (Cambridge, MA, USA, http://mim.forsyth.org). Labeled nucleotide Cy3-dCTP was incorporated during a second nested PCR. Next, hybridization was performed overnight at 55°C. The arrays were then washed at room temperature, spun dry, and stored in a dark container until they were scanned by the Axon 4000B microarray scanner, and crude data were extracted using GenePix Pro software (Molecular Devices, Sunnyvale, CA, USA). Microbial profiles were generated from image files of scanned arrays using a HOMIM online analysis tool (http://bioinformatics.forsyth.org/homim/). Detection of a particular taxon was determined by the presence of a fluorescent spot for that unique probe. The mean intensity for each taxon was calculated from hybridization spots for the same probe. Signals were normalized by comparison of individual signal intensities with the average of signals from universal 16S rRNA probes and categorized into relative intensity values ranging from 0 to 5 (the minimum threshold for signal detection is equivalent to approximately 10^4^ bacterial cells) [9,10,11].

### Statistical Analysis

The Chi-squared test was performed to compare the frequency of each probe between the SECC and caries-free groups. The nonparametric Mann–Whitney U test was performed to compare the abundance of species between the two groups. Adjustment for multiple comparisons was performed using the false-discovery rate (Benjamini and Hochberg, 1995). A p-value of ≤0.05 was considered statistically significant. Statistical analyses were performed using the SPSS 19.0 software.

## Results

The severe ECC and caries-free (control) groups were matched for age, gender, and race (Table 1). In total, 379 bacterial species were detected in both the saliva and supragingival plaque samples from all children. All collected samples were successfully analyzed.

**Fig 1:**
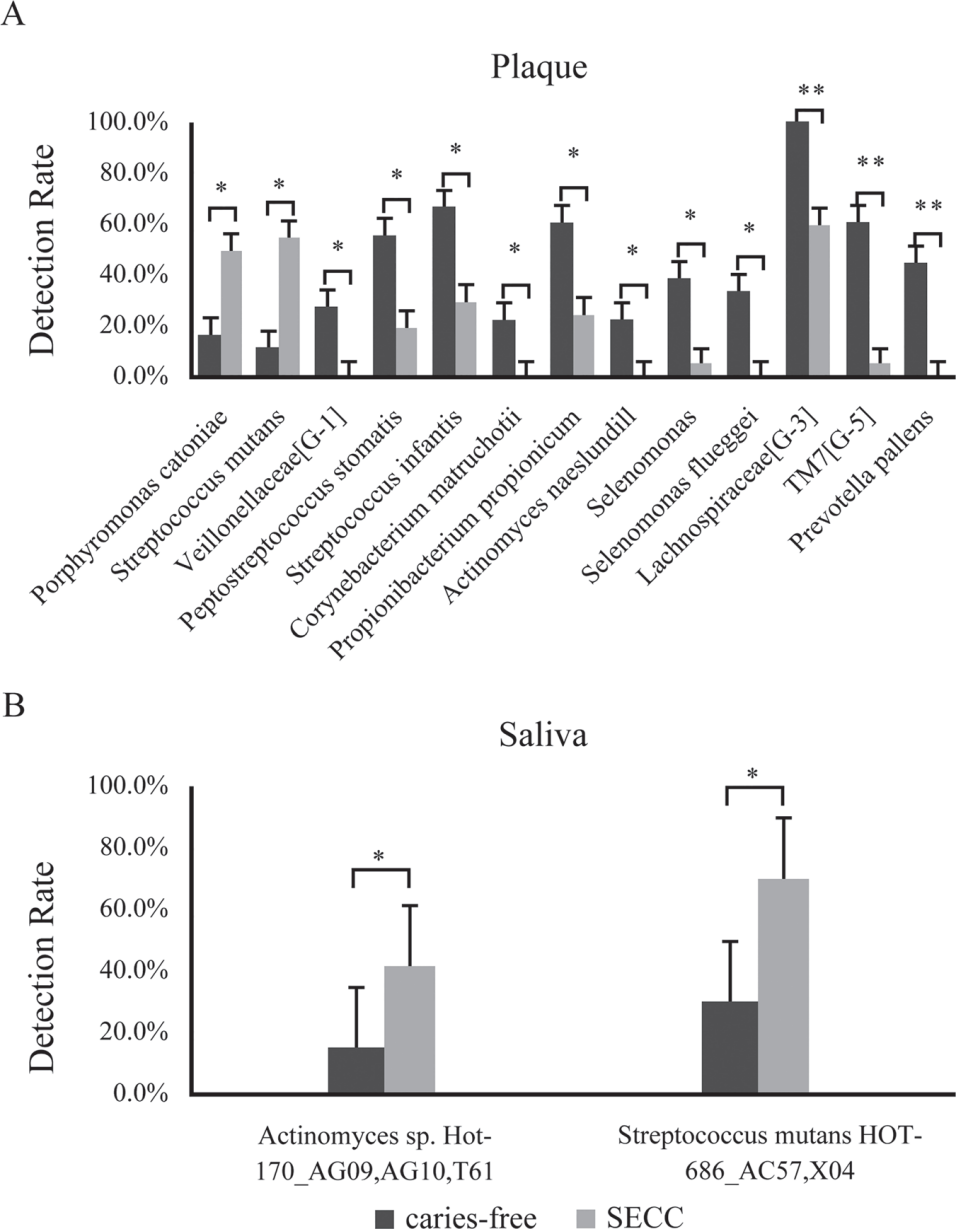
Bacterial species showing significant differences in prevalence between the severe ECC group and caries-free group. Significant differences in prevalence are observed for 13 bacterial species in supragingival plaque (A) and two bacterial species in saliva (B). *p ≤ 0.005, **p ≤ 0.05 according to the Chi-square test.

**Fig 2:**
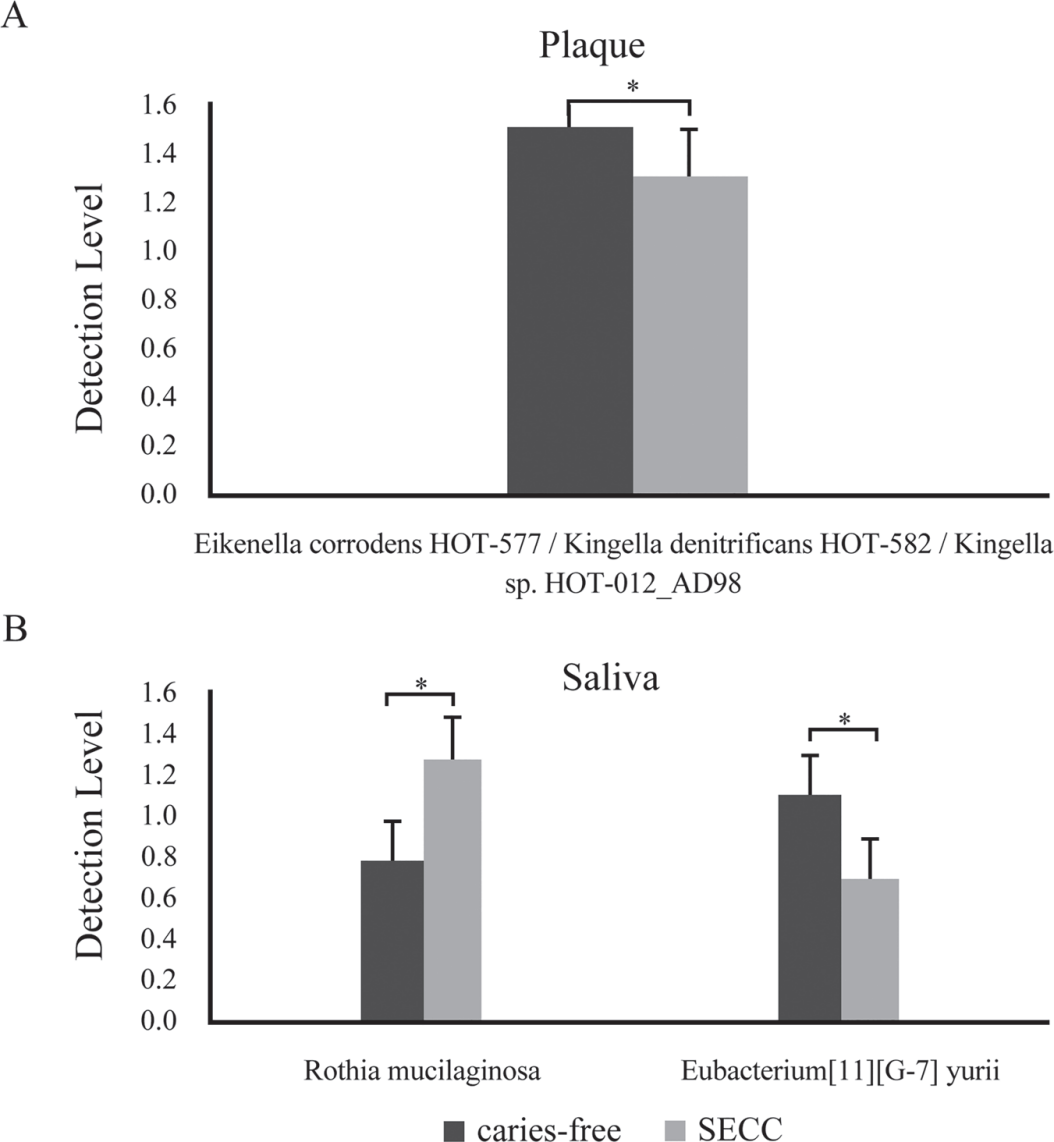
Bacterial species showing significant differences in abundance between the severe ECC group and caries-free group. One and three bacterial species showed significant differences in abundance in supragingival plaque (A) and saliva (B), respectively. *p<0.05 according to the Mann–Whitney Rank Sum Test.

Thirteen bacterial species showed significant differences in prevalence in supragingival plaque between the SECC and caries-free groups (Fig 1A), while two bacterial species showed significant differences in prevalence in saliva (Fig 1B). With regard to abundance, one bacterial species, *Eikenella corrodens* HOT-577/*Kingella denitrificans* HOT-582/*Kingella* sp. HOT-012_AD98, in supragingival plaque (p = 0.014, Mann–Whitney U test; Fig 2A) and two bacterial species, *Rothia mucilaginosa* (p = 0.046, Mann–Whitney U test) and *Eubacterium* (p = 0.021, Mann–Whitney U test), in saliva (Fig 2B) showed significant differences between the SECC and caries-free groups. Of these species, *S*. *mutans* was detected significantly more frequently in the SECC group than in the caries-free group in both saliva (p = 0.026, Chi-squared test) and plaque (p = 0.006, Chi-squared test). In addition, *Porphyromonas catoniae* and *Actinomyces* in plaque (p = 0.043, Chi-squared test) and saliva (p = 0.041, Chi-squared test), respectively, were more prevalent in the SECC group than in the caries-free group. *R*. *mucilaginosa* was more abundant in the saliva of children with SECC than in that of caries-free children.

Each of the 17 species was confirmed to be somehow relevant to the development of ECC or dental caries (Table 2). Table 3 summarizes the bacterial counts for each species.

**Table 2:** Bacterial species showing significant differences between the severe early childhood caries (SECC) and caries-free groups.

Significant differences in prevalence in plaque	P value	References	Opinions
*Actinomyces naeslundii*	0.041 ^a^	Beker et al.2002 ^[^ ^14^ ^,^ ^15^ ^]^	Associated with SECC
*Corynebacterium matruchotii*	0.041 ^a^	Tanner et al.2011 ^[^ ^1^ ^,^ ^19^ ^]^	Associated with oral health
*Lachnospiraceae* [G-3]	0.003 ^a^	Gross et al.2010 ^[^ ^19^ ^]^	Associated with SECC
*Porphyromonas catoniae*	0.043 ^a^	Vercher et al.2014 ^[^ ^17^ ^]^	Caries-related
*Prevotella pallens*	0.001 ^a^	Tanner et al.2011 ^[^ ^1^ ^]^	Associated with SECC
*Propionibacterium propionicum*	0.047 ^a^	Gross et al.2010 ^[^ ^19^ ^]^	Associated with SECC
*Selenomonas* spp.	0.016 ^a^	Xu et al.2012 ^[^ ^20^ ^]^	Associated with oral health
*Selenomonas flueggei*	0.007 ^a^	Luo et al.2012 ^[^ ^12^ ^]^	Caries-related
*Streptococcus infantis*	0.05 ^a^	Gross et al.2010 ^[^ ^19^ ^]^	Associated with oral health
*Streptococcus mutans*	0.006 ^a^	Li et al.2007 ^[^ ^2^ ^,^ ^3^ ^]^	Caries Pathogens
TM7 [G-5]	0.002 ^a^	Vercher et al.2014 ^[^ ^17^ ^]^	Caries-related
*Peptostreptococcus stomatis*	0.042 ^a^	Colombo et al.2009 ^[^ ^9^ ^]^	Associated with oral periodontitis
*Veillonellaceae* [G-1]	0.017 ^a^	Tanner et al.2012 ^[^ ^25^ ^]^	Associated with white-spot lesions
**Significant differences in abundance in plaque**	**P value**	**References**	**Opinions**
*Kingella denitrificans*	0.014 ^b^	Torlakovic et al.2012 ^[^ ^7^ ^]^	Associated with oral health
**Significant differences in prevalence in saliva**	**P value**	**References**	**Opinions**
*Actinomyces*	0.041 ^a^	Beker et al.2002 ^[^ ^14^ ^,^ ^15^ ^]^	Associated with SECC
*Streptococcus mutans*	0.026 ^a^	Li et al.2007 ^[^ ^2^ ^,^ ^3^ ^]^	Caries Pathogen
**Significant differences in abundance in saliva**	**P value**	**References**	**Opinions**
*Eubacterium*/ *Peptostreptococcaceae*	0.021 ^b^	Gross et al.2010 ^[^ ^19^ ^]^	Associated with oral health
*Rothia mucilaginosa*	0.046 ^b^	Colombo et al.2009 ^[^ ^9^ ^]^	Associated with peritonitis

The table shows 17 bacterial species that showed significant differences between the caries-free and SECC groups The studies showed that each species was somehow related to early childhood caries or dental caries.

^a^ Chi-squared test

^b^ Nonparametric Mann–Whitney U test

**Table 3:** Human Oral Microbe Identification Microarray (HOMIM) score distribution for the 17 bacterial species that showed significant differences between the severe early childhood caries (SECC) and caries-free groups.

	HOMIM scores									
Bacterial species	Severe ECC n = 20					Caries-free n = 18/ 20				
	≤1	2	3	4	5	≤1	2	3	4	5
*Actinomyces naeslundii II*	20	0	0	0	0	17	1	0	0	0
*Corynebacterium matruchotii*	20	0	0	0	0	18	0	0	0	0
*Lachnospiraceae* [G-3]	12	8	0	0	0	2	14	2	0	0
*Porphyromonas catoniae*	14	6	0	0	0	17	1	0	0	0
*Prevotella pallens*	20	0	0	0	0	15	3	0	0	0
*Propionibacterium propionicum*	19	1	0	0	0	14	4	0	0	0
*Selenomonas*	20	0	0	0	0	12	6	0	0	0
*Selenomonas flueggei*	20	0	0	0	0	17	1	0	0	0
*Streptococcus infantis*	18	2	0	0	0	17	1	0	0	0
*Streptococcus mutans* (in plaque)	12	3	3	1	1	17	1	0	0	0
TM7[G-5]	19	1	0	0	0	12	6	0	0	0
*Peptostreptococcus stomatis*	18	2	0	0	0	11	7	0	0	0
*Veillonellaceae* [G-1]	20	0	0	0	0	16	2	0	0	0
*Kingella denitrificans*	9	11	0	0	0	9	8	0	1	0
*Actinomyces*	17	3	0	0	0	20	0	0	0	0
*Streptococcus mutans* (in saliva)	13	2	5	0	0	18	2	0	0	0
*Eubacterium/ Peptostreptococcaceae*	16	4	0	0	0	13	7	0	0	0
*Rothia mucilaginosa*	11	7	2	0	0	15	5	0	0	0

The table shows the bacterial counts obtained by HOMIM for each species that are significantly different between the SECC and caries-free groups (ranging from 0 to 5, the minimum threshold for signal detection is equivalent to approximately 10^4^ bacterial cells).

At the genus level, the most abundant bacterial genera detected in both the saliva and plaque samples from the SECC group were *Streptococcus*, *Actinomyces*, *Campylobacter*, *Capnocytophaga*, *Fusobacterium*, *Gemella*, *Haemophilus*, *and Neisseria*. Interestingly, these species were also the most abundant in the caries-free group (Table 4) as well as the most commonly detected genera in both groups (Table 5).We calculated the Simpson index to compare the difference in alpha-diversity between the SECC and caries-free groups, and no significant differences for both the saliva (p = 0.829, Mann–Whitney U test) and plaque (p = 0.101, Mann–Whitney U test) samples. Saliva exhibited a greater diversity in the overall microbial population compared with plaque in the caries-free group (p = 0.005, Mann–Whitney U test), but not in the SECC group (p = 0.33, Mann–Whitney U test).

**Table 4:** The most abundant species in the severe early childhood caries (SECC) and caries-free groups.

Bacterial species	Mean score	SD
**Most abundant species in saliva in the SECC group**		
*Streptococcus oralis*	4.1	0.6
*Haemophilus parainfluenzae*	3.7	1.0
*Neisseria*	2.7	1.3
*Fusobacterium*	2.6	0.6
*Cardiobacterium hominis*	2.5	0.5
**Most abundant species in plaque in the SECC group**		
*Fusobacterium*	3.3	0.9
*Haemophilus parainfluenzae*	3.3	1.2
*Capnocytophaga granulosa*	3.3	1.1
*Streptococcus oralis*	3.3	1.0
*Campylobacter gracilis*	2.9	0.9
*Cardiobacterium hominis*	2.7	0.7
**Most abundant species in saliva in the caries-free group**		
*Streptococcus oralis*	4.4	0.5
*Haemophilus parainfluenzae*	3.9	0.6
*Neisseria*	3.0	0.8
*Fusobacterium*	2.8	0.7
*Cardiobacterium hominis*	2.6	0.5
**Most abundant species in plaque in the caries-free group**		
*Fusobacterium*	3.3	0.7
*Capnocytophaga granulosa*	3.2	1.0
*Streptococcus oralis/Streptococcus sp*.	3.0	0.9
*Campylobacter gracilis*	2.9	0.9
*Cardiobacterium hominis*	2.8	0.8
*Haemophilus parainfluenzae*	2.8	1.0
*Fusobacterium periodonticum*	2.6	1.0

The table shows the most abundant bacterial species in the SECC group and the caries-free group. Abundance values are represented as the mean intensity value (from the Human Oral Microbe Identification Microarray-ranked signal scale of 0–5) for each group.

“Mean” indicates the mean abundance of species in the group

SD = standard deviation

**Table 5:** Species with the highest prevalances in the severe early childhood caries (SECC) and caries-free groups.

Bacterial species	Prevalence	Mean score
**Most frequent species in saliva in the SECC group**		
*Granulicatella adiacens*/*Granulicatella elegans*	96%	1.6
*Campylobacter concisus*/*Campylobacter rectus*	95%	2.3
*Streptococcus australis*	95%	3.3
*Streptococcus mitis*/*Streptococcus* sp.	95%	2.4
*Veillonella dispar*/*Veillonella parvula*	95%	2.0
**Most frequent species in plaque in the SECC group**		
*Actinomyces naeslundii*	95%	2.3
*Campylobacter gracilis*	95%	2.9
*Capnocytophaga granulosa*	95%	3.3
*Campylobacter showae*	95%	2.6
*Gemella haemolysans*	95%	3.3
*Haemophilus parainfluenzae*	95%	3.3
*Leptotrichia hofstadii/Leptotrichia* sp.	95%	2.2
*Lachnoanaerobaculum saburreum*	95%	2.1
*Streptococcus anginosus/Streptococcus gordonii*	95%	2.8
*Streptococcus constellatus/Streptococcus intermedius*	95%	2.7
*Streptococcus cristatus*	95%	2.7
*Veillonella dispar/Veillonella parvula*	95%	1.9
**Most frequent species in saliva in the caries-free group**		
*Abiotrophia defectiva*	100%	2.3
*Cardiobacterium hominis*	100%	2.6
*Fusobacterium*	100%	2.8
*Gemella haemolysans/Gemella sanguinis*	100%	4.3
*Rothia dentocariosa/Rothia mucilaginosa*	100%	2.0
*Streptococcus australis*	100%	3.5
*Campylobacter concisus/Campylobacter rectus*	100%	2.7
*Haemophilus parainfluenzae*	100%	3.9
*Neisseria*	100%	3.0
*Streptococcus constellatus/Streptococcus intermedius*	100%	3.2
*Streptococcus infantis*	100%	2.5
*Streptococcus mitis*	100%	2.5
*Streptococcus oralis*	100%	4.4
*Streptococcus salivarius/Streptococcus vestibularis*	100%	4.1
**Most frequent species in plaque in the caries-free group**		
*Campylobacter gracilis*	100%	2.9
*Campylobacter showae*	100%	2.6
*Capnocytophaga granulosa*	100%	2.8
*Cardiobacterium hominis*	100%	2.8
*Fusobacterium*	100%	3.3
*Gemella haemolysans*	100%	3.2
*Haemophilus parainfluenzae*	100%	2.8
*Neisseria*	100%	2.3
*Streptococcus oralis*	100%	3.0

The table shows the most frequently detected bacterial species in the SECC group and the caries-free group.

“Prevalence” is expressed as the percentage of all samples that showed a positive signal for the designated Human Oral Microbe Identification Microarray probe.

“Mean score” indicates the mean abundance of species in the group.

## Discussion

Dental caries is one of the most prevalent and cost-ineffective oral infectious diseases worldwide. The prevalence of caries in the primary dentition underlies the importance of its prediction and prevention. Furthermore, ECC has become a prevalent public health problem among preschool children worldwide. However, little is known about the microbial community in ECC. The detection of specific bacteria associated with ECC can facilitate the prevention and treatment of dental caries in young children.

Although several previous studies have focused on the oral microbiota of children with and without dental caries, our research is the first, as per our knowledge, to use HOMIM to determine the bacterial profiles in both saliva and supragingival plaque to investigate the etiology of SECC in the primary dentition. The data generated will help in defining the microbial diversity in saliva and plaque, identify potential biomarkers of SECC, and provide new insights into the disease as will be discussed below.

Our study confirmed differences in microbial profiles between caries-free children and children with SECC and identified several bacterial species that could play a destructive role in this disease. *S*. *mutans* was found to be strongly associated with ECC in both saliva and supragingival plaque, confirming the findings of previous reports concerning dental caries and ECC [1,2]. Other species associated with SECC included *P*. *catoniae*, *Actinomyces*, and *R*. *mucilaginosa*. The presence of these species in healthy children can represent a marker of ECC risk, facilitating the detection of this disease in healthy individuals.

HOMIM was also conducted in a similar study by Luo *et al*. [12], who collected saliva from 50 children aged 6–8 years old to determine differences in bacterial profiles between children with and without caries. They concluded that *Bacteroidetes*, *Capnocytophaga sputigena*, *Tannerella*, *Campylobacter showae*, *Selenomonas*, and *Parvimonas* were significantly more common in the active caries group (DMFS>8) than in the caries-free group; however, our saliva samples showed insignificant differences (p > 0.05) for these species between two groups.

*S*. *mutans* is strongly associated with SECC in numerous investigations using different methods [13]. As opposed to the finding of Luo *et al*., who detected *S*. *mutans* in only one sample from the active caries group [12], *S*. *mutans* was detected in 14 saliva and 11 plaque samples from the SECC group in our study, which was a significantly higher prevalence than that in the caries-free group.

*Actinomyces* was detected significantly more frequently in the saliva samples from the SECC group, whereas there were no differences between the two groups in Luo *et al*.’s study [12]. With most evidence linking the species to root surface caries, *Actinomyces* has also been suspected to play a role in ECC. Beker’s and Jiang’s teams both observed high mean levels of *Actinomyces* in the earliest stage of childhood caries [14,15]; it was speculated that *Actinomyces* may play a major role in the initial formation stage of caries.

Differences in the age groups of patients included in Luo *et al*.’s study may be the reason for the differences in results between our study and theirs, because the microbial composition of the deciduous dentition is not the same as that of the mixed dentition. Moreover, the bacterial lysis method employed in our study may have yielded a better representation of gram-positive and gram-negative organisms. Considering that gram-positive bacteria such as *Streptococcus* are difficult to lyse, particularly in plaque, we modified the lysis procedure. All our samples were treated with a cocktail lysis buffer containing mutanolysin, proteinase K, and a lysozyme, which led to high quality and increased concentration of the total bacterial genomic DNA and a wide 16S rRNA gene diversity [2].

*Lactobacillus* has also been consistently associated with caries and is believed to be an important secondary pathogen in dental caries [1,2,3]. Surprisingly, we rarely detected this genus in both the SECC and caries-free groups in our study, consistent with the findings of Luo *et al*. This interesting phenomenon needs further investigation.

A novel finding of the current study was the association of *P*. *catoniae* with SECC, which suggested this bacteria to be a potential risk factor for the disease. Known as periodontal pathogens, *P*. *catoniae* organisms are saccharolytic, gram-negative, anaerobic rods belonging to early colonizers among obligate anaerobes [16]. A recent study showed that children with a poor caries status possessed a significantly higher level of *Porphyromonas* in their saliva [17]. However, Crielaard *et al*. found a positive association between the signal of the probe targeting *P*. *catoniae* in caries-free children, and the abundance of this species correlated negatively with the DMFS score [26]. Fabris *et al*. discovered that *Porphyromonas* was predominant in the root canals of primary teeth with necrotic pulp and was associated with primary endodontic infections and acute periradicular abscesses [18]. However, further studies of *P*. *catoniae* and its correlation with ECC are required for clearer conclusions.

Another interesting finding in the present study pertained to health-associated species. Some species were more prevalent in the caries-free group and were present in larger quantities and proportions; these included *Actinomyces naeslundii II*, *Corynebacterium matruchotii*, *Eubacterium*, *Lachnospiraceae*, *Selenomonas*, *S*. *infantis*, *Veillonellaceae*, and *Propionibacterium propionicum*. These health-associated species help in maintaining the balance of the oral microbiota. Tanner *et al*. reported a higher prevalence of *C*. *matruchotii* and *Actinomyces Cluster 1* in the caries-free group than in the SECC group [1]. Gross *et al*. reported that the levels of *S*. *infantis*, *C*. *matruchotii*, *Eubacterium* IR009, and *Lachnospiraceae* sp. C1 showed a significant decrease with caries progression [19]. Xu *et al*. found *Selenomonas* in supragingival plaque samples from caries-free children [20].

In our study, saliva exhibited a greater diversity in the overall microbial population compared with plaque in the caries-free group. The opposite was true for the SECC group, although the difference was not statistically significant (p = 0.33). Furthermore, there were significant differences between saliva and plaque in the prevalence and abundance of species. Considerable differences in bacterial composition and diversity between individual sites and surfaces in the oral cavity have been demonstrated [21]. Previous checkerboard hybridization and 16S rDNA sequencing studies have confirmed that the salivary flora is more similar to tongue flora than to dental plaque flora [22]. Some studies have found an association between microbiota and disease in plaque samples, but not in saliva samples, from patients with both gingivitis and dental caries [23,24].

Major limitations of the current study was the lack of identification of the underlying mechanism and lack of adequate follow-up for both groups; therefore, the development of fresh caries in high-risk children was not monitored and the microbiota in pre- and post-treatment samples from children with SECC was not compared.

A confirmatory study and a follow-up of 6–12 months would enable the determination of more specific associations between some of these species and SECC. Such a study will also lead to an increased understanding of the role of these bacteria in the initiation and progression of ECC and facilitate the prevention and treatment of dental caries in young children.

## Supporting Information

S1 Dataset The analysis result sent back from the Forsyth Institute. Signals were normalized by comparison of individual signal intensities with the average of signals from universal 16S rRNA probes and categorized into relative intensity values ranging from 0 to 5.
(XLS)

S1 Fig The original image result of microarray analysis. (TIF)

S1 Table The demographic data of all subjects. (XLS)
