# Supplementary figures and images for: Comparison of Oral Microbial Profiles between Children with Severe Early Childhood Caries and Caries-Free Children Using the Human Oral Microbe Identification Microarray
Source: PLoS One. 2015 Mar 30;10(3):e0122075. doi: 10.1371/journal.pone.0122075 (PMC4378984; doi:10.1371/journal.pone.0122075)

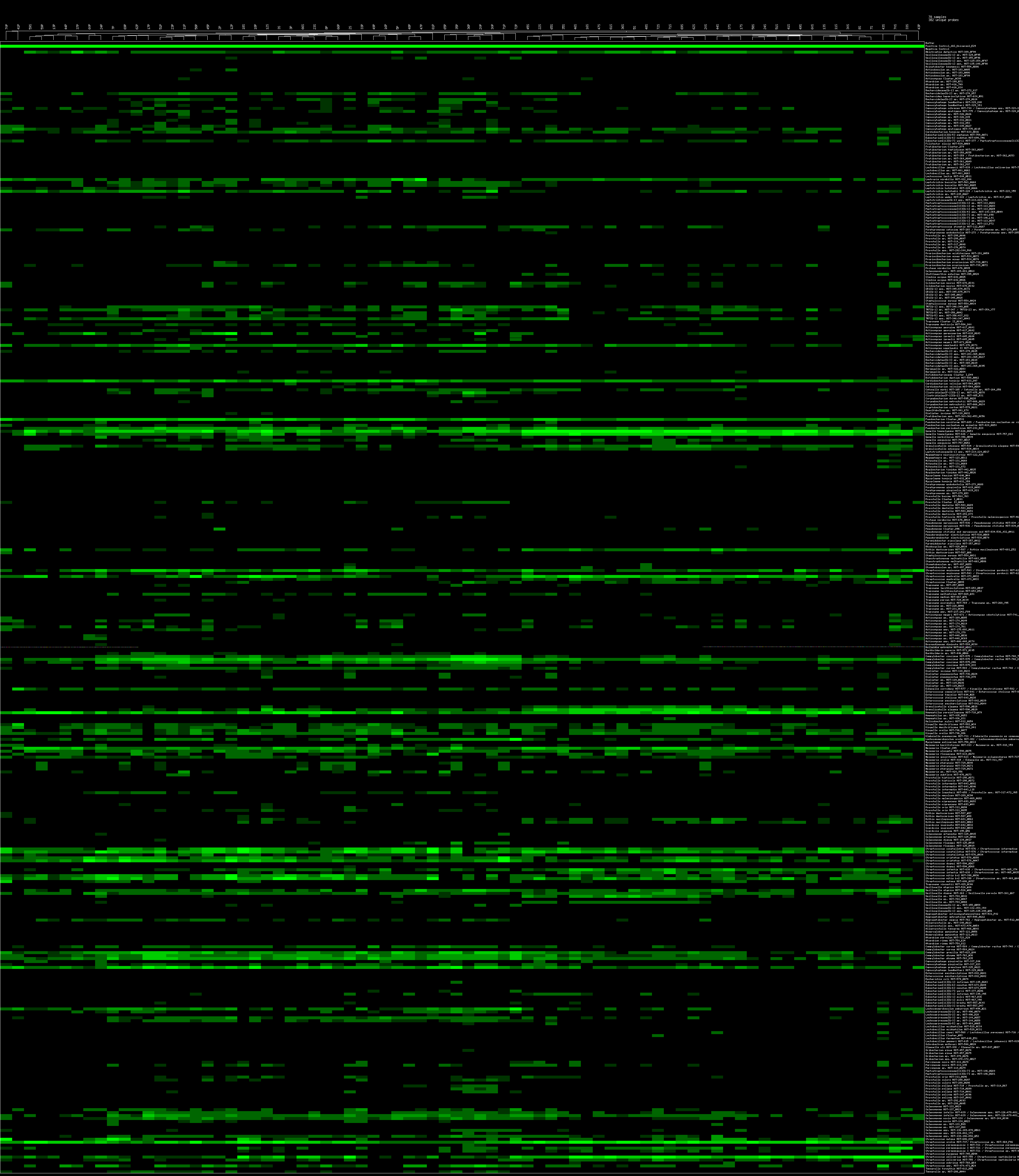

Supplement: S1 Fig — (TIF) [file pone.0122075.s002.tif]
